# Data on hydroxychloroquine interference with urine laboratory testing

**DOI:** 10.1016/j.dib.2019.104781

**Published:** 2019-11-08

**Authors:** Jennie M. Kingery, Joshua B. Radke, Jon Maakestad, Matthew D. Krasowski

**Affiliations:** a Department of Pathology, University of Iowa Hospitals and Clinics, 200 Hawkins Drive, Iowa City, IA, 52242, USA; b Department of Emergency Medicine, University of Iowa Hospitals and Clinics, 200 Hawkins Drive, Iowa City, IA, 52242, USA

**Keywords:** Absorbance error, Assay interference, Clinical chemistry tests, Drug of abuse testing, Hydroxychloroquine, Photometry

## Abstract

Hydroxychloroquine is a medication used to treat rheumatoid arthritis, systemic lupus erythematosus, and other autoimmune disorders. Previous studies have shown that hydroxychloroquine and the structurally related drug chloroquine have the potential to interfere with some common urine chemistry tests, especially at high concentrations. In the related research article, we observed suspected interference with urine drug of abuse testing in a patient who ingested approximately 12 g of hydroxychloroquine in an acute overdose, with urine hydroxychloroquine concentrations exceeding 500 mg/L. This case prompted a more detailed investigation of the effects of hydroxychloroquine spiked into pooled de-identified urine specimens from a hospital clinical laboratory. The data in this article provides the raw data for 24 urine assays that were investigated. The analyzed data is provided in the tables included in this article. The dataset reported is related to the research article entitled “Diagnostic Pitfalls and Laboratory Test Interference After Hydroxychloroquine Intoxication: A Case Report” [1].

**Table:** Specifications Table

Subject	Medicine and Dentistry
Specific subject area	Pathology and Medical Technology
Type of data	Table Figure
How data were acquired	Urine chemistry assays run on Roche Diagnostics cobas c501, c502, e602, and c701 clinical chemistry analyzers
Data format	Raw and Analyzed
Parameters for data collection	Pools of de-identified urine specimens from the hospital clinical laboratory were analyzed without hydroxychloroquine and with hydroxychloroquine (Sigma-Aldrich, St. Louis, MO) spiked at concentrations of 1, 10, 100, 500, and 1000 mg/L.
Description of data collection	Data for 13 of the assays (amylase, calcium, chloride, creatinine, glucose, human chorionic gonadotropin, magnesium, NGAL, pH, phosphorus, potassium, protein, and sodium) were analyzed by analysis of a single urine pool. Data for 11 of the assays (amphetamines screen, benzodiazepines screen, buprenorphine screen, cocaine metabolite screen, cotinine screen, microalbumin, myoglobin, opiates screen, oxycodone screen, urea nitrogen, and tetrahydrocannabinol screen) were analyzed by analysis of four separate urine pools.
Data source location	Iowa City, Iowa, United States of America
Data accessibility	With the article
Related research article	Author's name Joshua B. Radke, Jennie M. Kingery, Jon Maakestad, Matthew D. Krasowski Title Diagnostic Pitfalls and Laboratory Test Interference After Hydroxychloroquine Intoxication: A Case Report Journal Toxicology Reports [1] DOI: 10.1016/j.toxrep.2019.10.006

**Table:** 

**Value of the Data** • The data provided is of value as there is currently only limited published data demonstrating interfering effects of hydroxychloroquine on urine laboratory assays. • Other researchers or personnel in clinical laboratories might find this data useful as a reference for comparison. • Our data set would serve as a starting point for researchers interested in future investigations studying the effects of hydroxychloroquine on urine chemistry assays marketed by vendors. • The data is of value as previous studies have not examined a wide range of urine laboratory assays commonly performed in clinical laboratories. • The data provide information for 24 urine assays tested up to 1000 mg/L hydroxychloroquine, a concentration achievable in large overdose.

## 1 Data

We investigated the effect on urine assays of hydroxychloroquine at concentration up to 1000 mg/L spiked into pools of de-identified urine specimens from the university medical center central clinical laboratory. This followed from observation that a patient with a large overdose of hydroxychloroquine showed suspected interference for some urine laboratory tests obtained for clinical care [1]. There is limited published data that hydroxychloroquine can interfere with urine protein dipstick methods [2,3] and with some urine drug screening tests [4]. A detailed review of package inserts for drug of abuse and therapeutic drug monitoring assays did not find hydroxychloroquine reported as an interferent in any of the assays [5,6].

The 24 specific urine assays analyzed are as follows: amphetamines drug screen, amylase, benzodiazepines drug screen, buprenorphine drug screen, calcium, chloride, cocaine metabolite screen, cotinine screen, creatinine, glucose, human chorionic gonadotropin (hCG), magnesium, microalbumin, myoglobin, NGAL, opiates drug screen, oxycodone drug screen, pH, phosphorus, potassium, protein, sodium, tetrahydrocannabinol (THC) drugs screen, and urea nitrogen. Technical details on these 24 urine assays are available elsewhere [1]. The assays were initially screened in triplicate for a single de-identified pooled urine sample. Thirteen of the assays showed no absorbance or other alarms/errors and also did not have any results that differed by more than 15% from the control without hydroxychloroquine. The raw data for these 13 assays is in Table 1. Raw data for the remaining 11 assays is in Table 2. These were all tested in triplicate for a total of 4 separate pooled urine samples.

**Table 1:** Raw data for 13 urine assays on a single pooled urine samples.

Assay	Units	Hydroxychoroquine concentration					
		0 mg/L	1 mg/L	10 mg/L	100 mg/L	500 mg/L	1000 mg/L
Amylase	U/L	121	121	123	122	121	122
Calcium	mg/dL	10.4	10.4	10.6	10.8	9.9	10.0
Chloride	mmol/L	69.0	70.7	68.0	70.3	72.7	76.7
Creatinine	mg/dL	76.9	79.3	79.3	77.8	82.3	80.7
Glucose	mg/dL	6.0	5.5	5.4	5.3	6.0	6.0
hCG	mIU/mL	1.0	1.1	1.1	1.1	1.0	1.1
Magnesium	mg/dL	4.8	4.7	4.7	4.7	4.3	4.2
NGAL	ng/mL	12.0	11.0	11.5	12.0	11.0	11.5
pH	pH units	7	6.8	6.9	6.9	6.9	6.9
Phosphorus	mg/dL	20.4	21.3	22.0	21.6	19.6	20.1
Potassium	mmol/L	35	35	34	35	39	39
Protein	mg/dL	55	57	57	58	54	49
Sodium	mmol/L	60	62	61	62	65	66

**Table 2:** Raw data for 13 urine assays on four separate pooled urine samples.

Assay	Units	Sample #	Hydroxychloroquine concentration					
			0 mg/L	1 mg/L	10 mg/L	100 mg/L	500 mg/L	1000 mg/L

Amphetamines Drug Screen	Relative	1	−268	−286	−276	−281	−271	−250
	absorbance units	2	−302	−323	−309	−314	−298	−291
	(positive: >0)	3	−273	−283	−284	−279	−281	−264
		4	−299	−299	−295	−298	−283	−265
Benzodiazepines Drug Screen	Relative	1	−187	−178	−176	−174	−171	−164
	absorbance units	2	−263	−204	−209	−201	−185	−58
	(positive: >0)	3	−210	−181	−183	−176	−176	−172
		4	102	108	106	105	105	107
Buprenorphine Drug Screen	Relative	1	−339	−441	−441	−472	−534	Absorbance error
	absorbance units	2	−283	−421	−410	−429	−472	Absorbance error
	(positive: >0)	3	−283	−393	−398	−420	−418	Absorbance error
		4	−258	−389	−395	−405	−451	Absorbance error
Cocaine Drug Screen	Relative	1	−548	−538	−554	−557	−565	−540
	absorbance units	2	−575	−587	−570	−581	−578	−558
	(positive: >0)	3	−553	−543	−546	−546	−537	−536
		4	−558	−560	−568	−570	−551	−552
Cotinine Screen	Relative	1	>2000	>2000	>2000	>2000	>2000	Absorbance error
	absorbance units	2	1294	786	810	784	705	Absorbance error
	(positive: >0)	3	−250	−194	−182	−202	−268	Absorbance error
		4	229	398	397	348	119	Absorbance error
Microalbumin	mcg/mg creatinine	1	293	273	273	281	315	329
		2	155	105	105	112	144	189
		3	24	20	23	24	26	30
		4	215	225	224	231	249	274
Myoglobin	ng/mL	1	<21	<21	<21	<21	<21	<21
		2	1008	899	900	876	752	599
		3	<21	<21	<21	<21	<21	<21
		4	<21	<21	<21	<21	<21	<21
Opiates Drug Screen	Relative	1	88	103	101	101	96	89
	absorbance units	2	−520	−497	−496	−496	−469	−436
	(positive: >0)	3	−483	−477	−455	−475	−458	−459
		4	−479	−446	−453	−447	−426	−392
Oxycodone Drug Screen	Relative	1	−158	−159	−160	−158	−158	Absorbance error
	absorbance units	2	28	31	32	31	27	Absorbance error
	(positive: >0)	3	−154	−159	−159	−157	−156	Absorbance error
		4	−155	−155	−156	−155	−153	Absorbance error
Urea nitrogen	mg/dL	1	859	862	884	877	866	863
		2	684	696	692	694	673	647
		3	951	927	930	926	930	923
		4	541	529	531	519	519	518
THC Drug Screen	Relative	1	−178	−165	−162	−163	−155	−126
	absorbance units	2	−221	−191	−186	−180	−167	−152
	(positive: >0)	3	−225	−206	−206	−207	−195	−201
		4	−225	−210	−211	−212	−194	−180

**Fig. 1:**
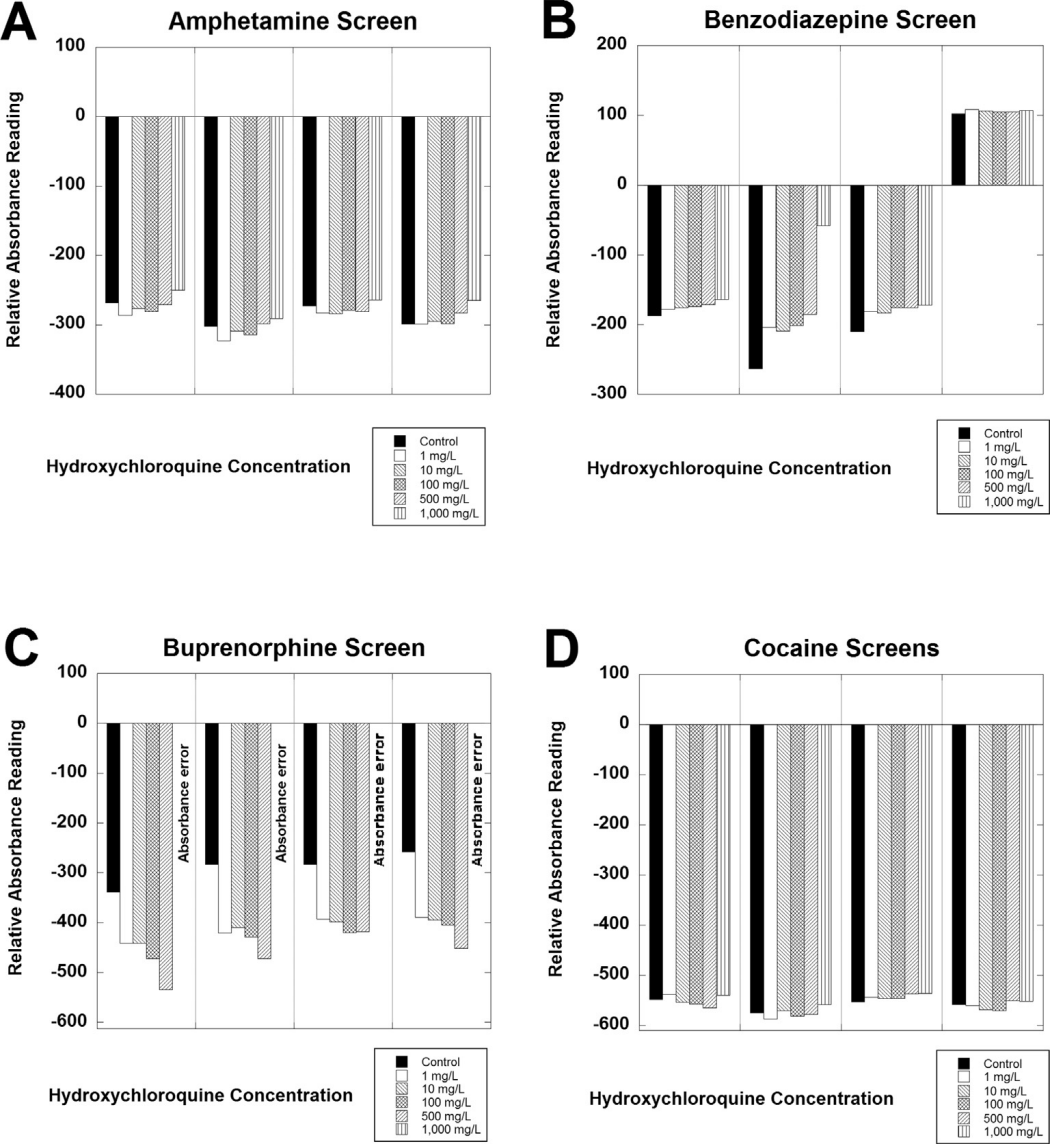
Analysis of hydroxychloroquine effects on urine assays for (A) amphetamine screen, (B) benzodiazepine screen, (C) buprenorphine screen, and (D) cocaine screen. Four separate de-identified pooled urine samples were tested in triplicate at the indicated hydroxychloroquine concentrations.

**Fig. 2:**
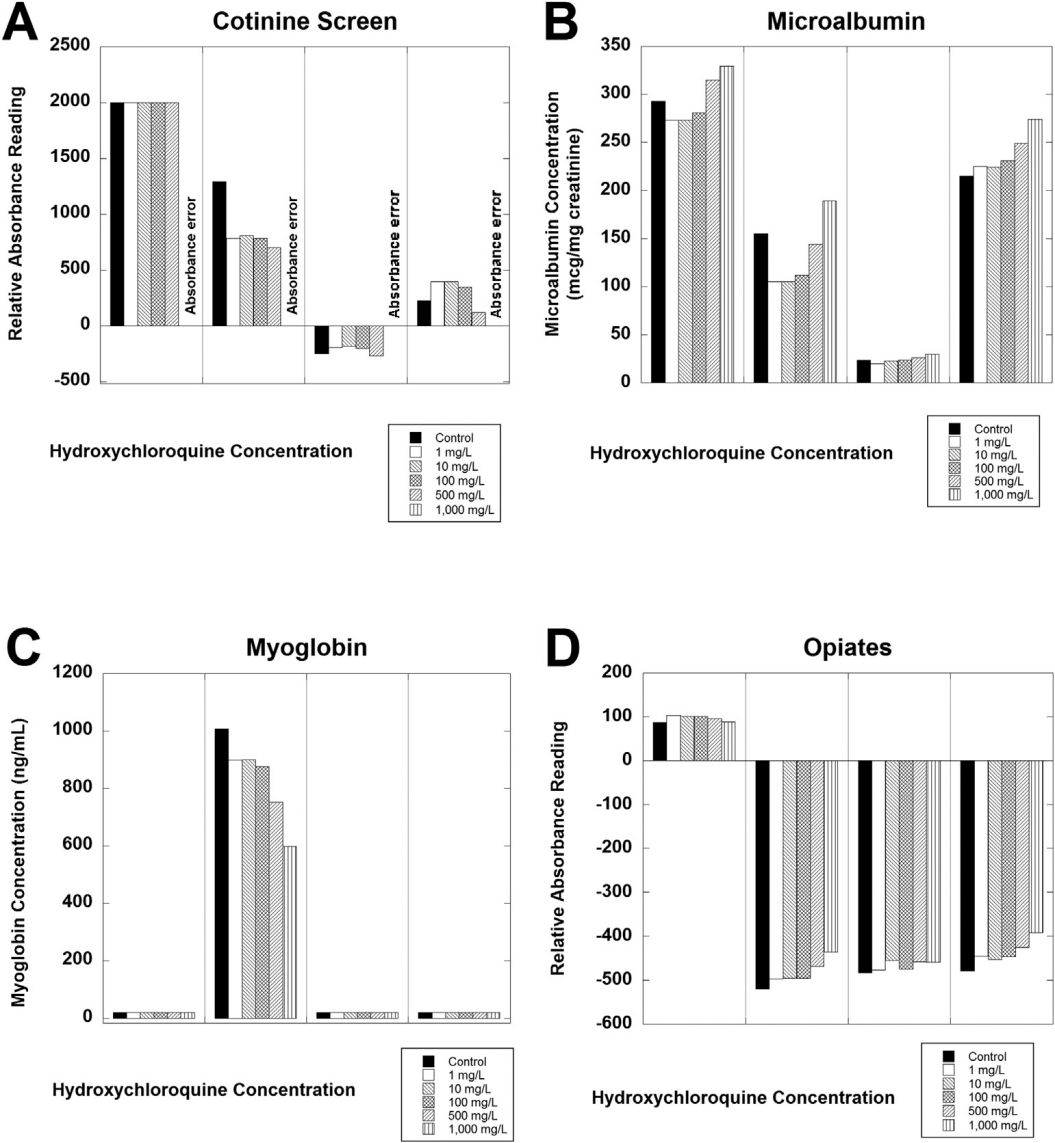
Analysis of hydroxychloroquine effects on urine assays for (A) cotinine screen, (B) microalbumin screen, (C) myoglobin assay, and (D) opiates screen. Four separate de-identified pooled urine samples were tested in triplicate at the indicated hydroxychloroquine concentrations.

**Fig. 3:**
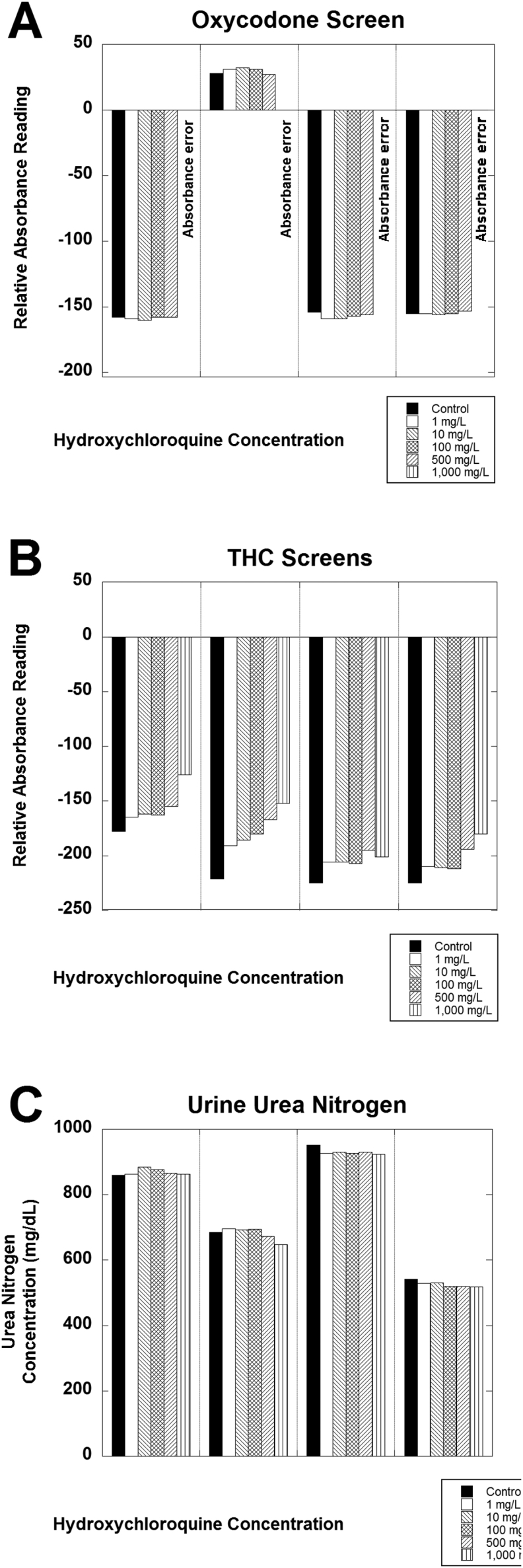
Analysis of hydroxychloroquine effects on urine assays for (A) oxycodone screen, (B) tetahydrocannabinol (THC) screen, and (C) urea nitrogen. Four separate de-identified pooled urine samples were tested in triplicate at the indicated hydroxychloroquine concentrations.

Fig. 1 shows data for 4 of the assays (amphetamine screen, benzodiazepine screen, buprenorphine screen, and cocaine screen). An absorbance alarm was evident for all 4 samples containing 1000 mg/L hydroxychloroquine for the buprenorphine screen (Fig. 1B). Fig. 2 shows data for another 4 of the assays (cotinine screen, microalbumin, myoglobin, and opiates screen). Positive bias and absorbance errors were evident for the cotinine assay for all 4 specimens (Fig. 2A). Biphasic effects of hydroxychloroquine were evident for the microalbumin assay, especially for samples 1, 2, and 4 (Fig. 2B). A negative bias was evident for the myoglobin assay for sample 2 (Fig. 2C). The remaining urine samples had <21 ng/mL myoglobin and did not show any evident effect of hydroxychloroquine, although a negative bias would not be detectable in these 3 samples with the myoglobin concentrations below the lower limit of quantitation. Fig. 3 shows data for the remaining 3 assays analyzed in detail. Absorbance errors were evident for the oxycodone for all 4 samples spiked with 1000 mg/L (Fig. 3A). A positive bias from hydroxychloroquine was evident for all 4 samples analyzed for tetrahydrocannabinol (THC; Fig. 3B), although all 4 of these samples would still be negative in this qualitative assay.

## 2 Experimental design, materials, and methods

All analyses were performed on Roche Diagnostics cobas 8000 analyzers (c501, c502, c602, and c701). The complete list of assays with vendor name, methodology, and assay version are summarized elsewhere [1]. All assays were run in accordance with package insert instructions. A total of 4 pooled urine specimens were prepared using de-identified specimens from the clinical laboratory. All 24 assays were tested in triplicate for 1 of the urine pools. As described above, 11 of the assays were then tested and triplicate for the remaining 3 urine pools. The raw data consists of concentration or absorbance units for the specimens. It should be noted that the urine total protein method used for the data in this report uses a biuret complex method using divalent copper in alikaline solution [7]. This is in contrast to previous studies using tetrabromophenol blue [3] and pyrogallol red-molybdate methods [2] that showed interference by hydroxychloroquine.
